# Carcinome épidermoide primitif du sein mimant un abcès

**DOI:** 10.11604/pamj.2018.30.84.15580

**Published:** 2018-05-29

**Authors:** Hafsa Chahdi, Babahabib Abdellah

**Affiliations:** 1 Service d’Anatomie Pathologique, Hôpital Militaire d’Instruction Mohamed V Rabat, Maroc; 2 Service de Gynécologie Obstétrique, Hôpital Militaire d’Instruction Mohamed V Rabat, Maroc

**Keywords:** Carcinome épidermoïde primitif, sein, carcinome métaplasique, Squamous cell carcinoma, breast, metaplastic cancer

## Abstract

Squamous cell carcinoma of the breast is a basal-like phenotype metaplastic cancer with poor prognosis. It is often diagnosed at earlier stages based on voluminous palpable mass not detected on screening. Squamous cell carcinoma should be suspected in patients with abscess, as is the case with our 44-year old female patient presenting with a mass in the left breast diagnosed as a cyst with abscess formation measuring 5,4cm x 5cm on mammography combined with ultrasound (A). Surgical biopsy was performed. Anatomopathological examination showed tumor proliferation with large carcinomatous lobules and clear squamous differentiation greater than 90% (B). Tumor cells were very atypical and showed many mitotic figures. Immunohistochemical study showed tumor cells expressing cytokeratins 5/6 (C). Proliferation index was 85% (D). Hormone receptors and hercept test were negative. The diagnosis of metaplastic squamous cell carcinoma was retained. Our patient underwent left mastectomy with axillary dissection and adjuvant chemotherapy followed by radiotherapy. The patient didn't developed locoregional recurrence or distant metastasis during a 9-month follow-up period.

## Image en médecine

Les cancers du sein de type épidermoïde appartiennent au cancer de type métaplasique, avec un pronostic défavorable se rapprochant des formes de phénotype basal. Son diagnostic est souvent fait au stade de masse palpable volumineuse, échappant aux campagnes de dépistage. On saura se méfier des abcès survenant chez certaines patientes comme c'est le cas de notre patiente âgée de 44ans, qui a consulté pour une masse du sein gauche diagnostiquée à la mammogr0aphie couplée à l'échographie comme une formation kystique d'allure abcédée mesurant 5,4cm x 5cm (A). Une biopsie chirurgicale a été réalisée et dont l'examen anatomopathologique trouvait une prolifération tumorale faite de larges lobules carcinomateux avec une différenciation malpighienne nette estimée à plus de 90%(B). Les cellules tumorales étaient très atypiques et montraient de nombreuses figures mitotiques. A l'étude immunohistochimique les cellules tumorales exprimaient les cytokératines 5/6 (C). L'indexe de prolifération était estimé à 85% (D). Les récepteurs hormonaux et l'hercept test étaient négatifs. Le diagnostic retenu était celui d'un carcinome métaplasique de type carcinome épidermoide. Notre patiente a bénéficié d'une mastectomie gauche avec curage axillaire et d'une chimiothérapie adjuvante suivie de radiothérapie. L'évolution avec un recul de neuf mois n'a pas révélé de récidive ou de métastase.

**Figure 1:**
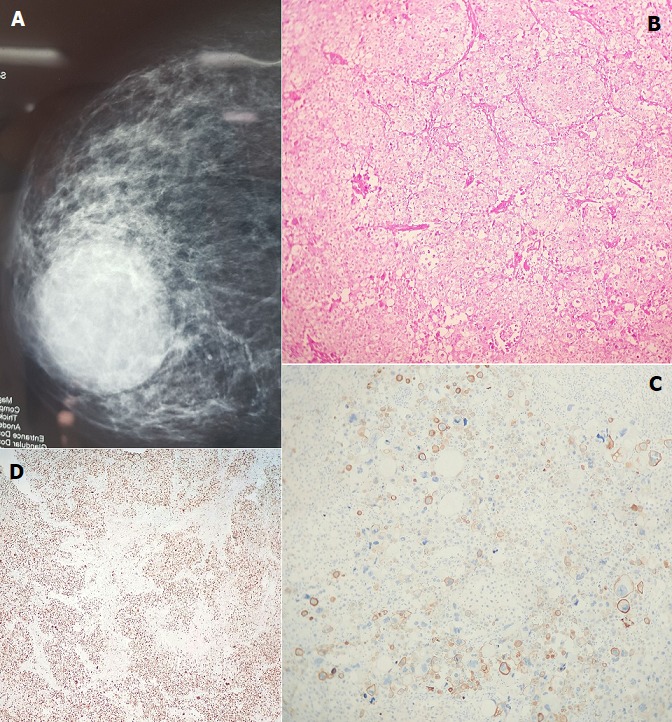
(A) image mammographique: masse kystique d’allure abcédée; (B) prolifération carcinomateuse à différenciation malpighiènne (Hemateine Eosine GX20); (C) immunohistochimie: marquage positif des cellules tumorales par la cytokératine 5/6; (D) immunohistochimie: marquage nucléaire de plus de 85% des cellules tumorales par le KI67 (indexe de prolifération)

